# Isolated rectal buttonhole tears in obstetrics: case series and review of the literature

**DOI:** 10.1007/s00192-020-04502-2

**Published:** 2020-09-15

**Authors:** Joanna C. Roper, Ranee Thakar, Abdul H. Sultan

**Affiliations:** 1 Department of Obstetrics and Gynaecology, Croydon University Hospital, London Road, Croydon, CR7 7YE UK; 2 St George’s University of London, London, UK

**Keywords:** Isolated rectal buttonhole tear, Rectovaginal fistula, Obstetric anal sphincter injury, Third and fourth degree tears, Rectal examination

## Abstract

**Introduction and hypothesis:**

The management of isolated rectal buttonhole tears is not standardised and can be challenging in an acute obstetric setting. Our aim was to review the published literature and describe management and repair techniques in a case series.

**Methods:**

A literature search was carried out. All results were screened and reviewed. Rectal buttonhole tears following vaginal delivery between April 2012 and January 2020 in our institution were identified. Repair technique and post-operative management were recorded.

**Results:**

There were nine published case reports (four instrumental deliveries, two vaginal breech and three normal vaginal deliveries). Four case reports described a two-layer closure and five described a three-layer closure. Two cases were repaired in collaboration with colorectal surgeons. All nine cases made an uneventful recovery. We identified three patients with buttonhole tears all of whom had instrumental deliveries. A colorectal surgeon repaired the tear in two layers in one case, and an obstetrician performed the repair in the other two cases, one in three layers and the other in two layers. One patient had a de-functioning stoma at a later date due to a second breakdown of the recto-vaginal fistula repair.

**Conclusion:**

Buttonhole tears are rare but techniques of repair vary. Most cases reviewed had an uneventful recovery after repair. We provide standardised steps for repair and management of isolated rectal buttonhole tears along with a video demonstrating the repair technique in an animal tissue (pig) model.

**Electronic supplementary material:**

The online version of this article (10.1007/s00192-020-04502-2) contains supplementary material, which is available to authorized users.

## Introduction

**Fig. 1:**
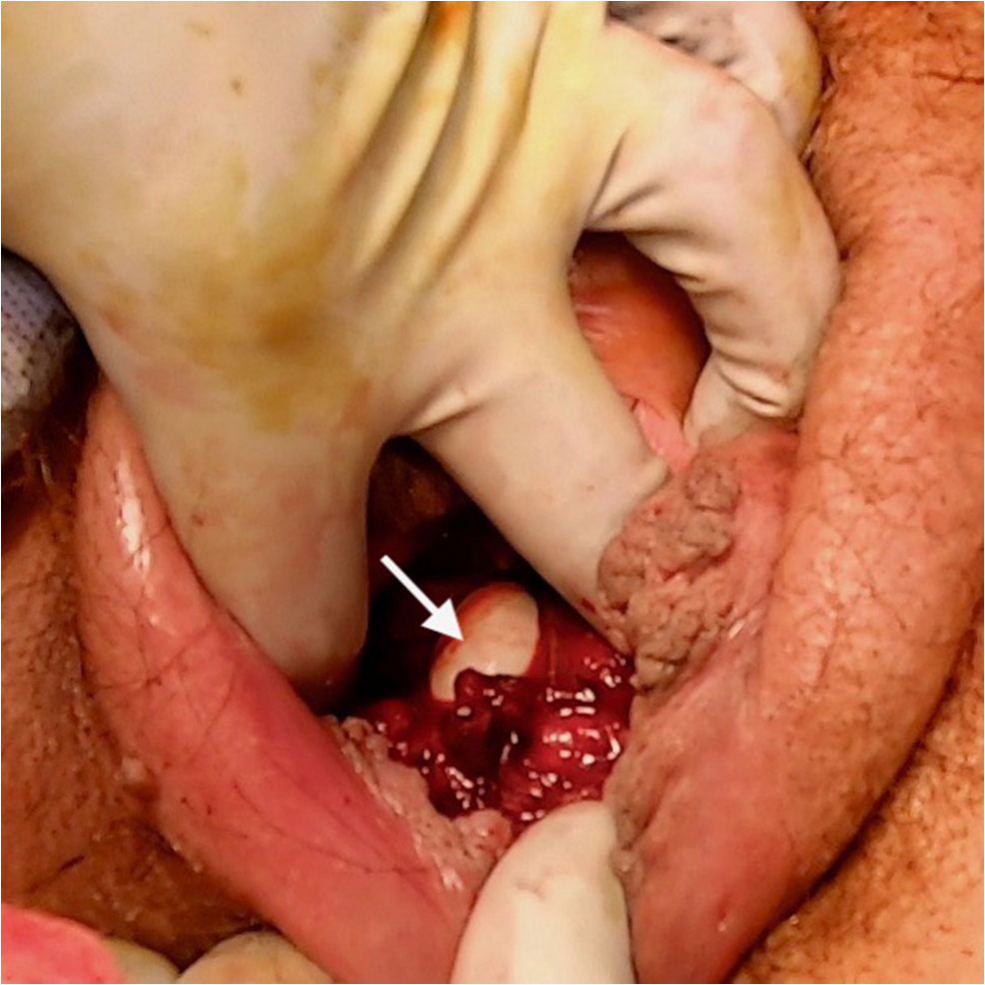
Isolated rectal buttonhole tear (arrow)

A rectal buttonhole tear is an isolated tear of the anal epithelium or rectal mucosa and vagina but without involving the anal sphincter [1]. It is not part of the widely accepted Sultan classification of perineal and anal sphincter trauma [2]. By definition, it is not a fourth-degree tear because the anal sphincter muscles are not torn and therefore should not be labelled as such. A buttonhole tear is rare, although its true incidence has not been reported [3]. It can occur concurrently with an obstetric anal sphincter injury (OASI), i.e. an isolated rectal tear occurs in conjunction with a separate tear involving the anal sphincter. However, this is extremely rare. Usually, the presentation is with an intact perineum at delivery or a first/second-degree tear, and consequently it can remain undiagnosed, without a structured and careful combined vaginal and rectal examination (Fig. 1). If unrecognised and therefore unrepaired, a rectovaginal fistula can persist [4, 5].

This type of injury is rare in humans but is commonly described in veterinary (mostly equine) case reports [6, 7]. Successful repair of these injuries is known to be particularly difficult because the anovaginal septum is a thin, poorly vascularised structure with faecal matter constantly passing it [8]. Furthermore, during defaecation and straining the pressure in the rectum is much higher and therefore can potentially predispose to disruption of the repair, highlighting the importance of a good repair technique.

Given their location, rectal mucosal tears are susceptible to infection and consequently a wound breakdown can lead to fistula formation. A recto-vaginal fistula leads to passive faecal soiling through the vagina and passage of vaginal flatus. These are devastating symptoms for a woman to endure and can have a dramatic effect on quality of life [9].

There are only a few case reports describing rectal buttonhole tears, with notable inconsistency in repair techniques [3, 6, 10–13]. Historically, there have been considerable variations in the techniques used to repair perineal and anal sphincter trauma in obstetrics [14]. A survey carried out by Fernando et al. [15] found that 30% of coloproctologists would recommend a covering colostomy for third- or fourth-degree tears. None of the obstetricians in that survey stated that they would request a colostomy for the same procedure. It is widely accepted in obstetrics that repair of perineal trauma, including rectal buttonhole tears, is usually by primary repair [4, 16].

Given the rarity of this condition, we first report on a short case series of isolated rectal buttonhole tears, their repair techniques and post-operative outcomes. Second, we describe a standardised technique to identify rectal buttonhole tears and perform an optimal repair.

## Materials and methods

This is a case series reviewing all published case reports of isolated rectal buttonhole tears after vaginal delivery and all cases from Croydon University Hospital between April 2012 to January 2020. A literature search of Medline, Cinahl and Emcare Cochrane Library, Trip database, BMJ Best Practice, BMJ Case Reports and Up-To-Date was conducted, using the MeSH terms: obstetric, isolated rectal tear, buttonhole rectal tear and vaginal delivery. All titles and abstracts were screened and relevant articles reviewed. Only case reports and case series were included. Data from Croydon University Hospital, a district general hospital, with a tertiary referral specialist perineal trauma and pelvic floor reconstruction unit, were obtained from review of case notes. Intra-operative details, post-operative management and outcomes were recorded from published case reports. A case series combined with personal experience of the authors has been used to produce a recommended protocol for repair of buttonhole tears (Table 3) accompanied with a video demonstration. (Video 1).

## Results

### Review of literature

One case report relating to an isolated rectal buttonhole tear during vaginal delivery was found from the literature search with the MeSH terms [10]. A further eight cases were identified in a hand search [3, 6, 12, 13, 17]. Therefore, in total, we included nine cases in our series (Table 1).

**Table 1:** Case reports of rectal buttonhole tears from the literature

Paper	Mode of delivery	Type of injury	Type of repair	Repair conducted by	Post-operative management	Follow-up and patient symptoms
Thirumagal, 2007 [10]	Normal vaginal delivery	6 cm vertical tear in posterior vaginal wall involving the rectum. Sphincter intact	Rectal mucosa and muscularis-Vicryl 1 continuous Vaginal wall- Vicryl rapide 2–0	Rectum-colorectal surgeon Vagina-obstetrician	Antibiotics and laxatives	3 months follow-up, asymptomatic. Endoanal ultrasound normal
Byrne, 2006 [6]	Ventouse delivery, occiput transverse (OT) position, with episiotomy	Episiotomy, 3a tear and 5 cm longitudinal tear in anterior rectal wall	Rectal mucosa, musculofibrous perineal body and vaginal mucosa-0 polyglactin (Vicryl). Perineum-undyed 2–0 polyglactin	Obstetrician with colorectal opinion	Low fibre diet, erythromycin, metronidazole and aperients	6 weeks follow-up, asymptomatic
Shaaban, 2008 [12]	Ventouse delivery, occiput anterior (OA) position, no episiotomy	4 cm midline, longitudinal rectovaginal tear, intact perineum	Rectal mucosa-interrupted Vicryl, with knots in the rectal lumen Vagina-continuous Vicryl	Obstetric consultant	Intravenous metronidazole and cefuroxime intra-op and 4 days post op. Light diet from day 3. Lactulose and ispaghula husk	6 weeks follow-up, asymptomatic
Vergers-Spooren, 2011 [3]	Breech vaginal delivery	2–3 cm of rectovaginal septum, 1 cm cranial of the anal canal and episiotomy	Rectal mucosa and rectovaginal septum-interrupted Monocryl 4–0 Vaginal mucosa- continuous Vicryl 2–0 ‘Routine’ episiotomy repair	Obstetrician	Augmentin intravenous intra-op. Magnesium oxide as stool softener	6 weeks follow-up and 3 months follow-up, asymptomatic. Endoanal ultrasound normal
Morrel, 1996 [13]	Ventouse delivery, with episiotomy	Episiotomy and midline 4 cm rectal lesion	Fourth-degree recto-vaginal lesion created by extending the episiotomy. Rectal mucosa repaired with atraumatic inverting sutures. Then anal sphincter, vagina and perineum repaired	Not reported	Antibiotics and laxatives were given	No complications. Asymptomatic of fistula
Morrel, 1996 [13]	Ventouse delivery, with episiotomy	Episiotomy and longitudinal 4 cm recto-vaginal lesion	Extending episiotomy to lesion, leaving anal sphincter intact. Rectal tear repaired using inverted sutures. Vagina and episiotomy repaired	Not reported	Antibiotics and laxatives given	Uncomplicated recovery, asymptomatic at follow-up
Morrel, 1996 [13]	Normal vaginal delivery	Midline 5 cm recto-vaginal lesion, perineum intact	2nd-degree surgical lesion created to improve visibility. Rectal mucosa repaired with inverting sutures. Vagina and perineum repaired	Not reported	Antibiotics and laxatives given	Uncomplicated recovery
Morrel, 1996 [13]	Normal vaginal delivery	3 cm recto-vaginal lesion, intact perineum	Rectum repaired with continuous inverting suture. Perirectal tissue repaired, levator ani strengthened with sutures	Not reported	Antibiotics and laxatives given	Uncomplicated follow-up
Diepenhorst, 2012 [17]	Breech vaginal delivery	Rectovaginal septum torn. Median episiotomy performed which extended to create 4th degree tear	Repaired as fourth degree tear. Rectal mucosa- interrupted 3-0 Vicryl. Reinforcing interrupted sutures in perirectal fascia. Vaginal mucosa- continuous inverting 3-0 Vicryl.	Not reported	Not reported	Uncomplicated follow-up

Three were normal vaginal deliveries, two breech vaginal deliveries and four ventouse-assisted deliveries. Four cases had a concurrent episiotomy, also repaired. One case had a concurrent 3a tear which was repaired separately [6]. Three cases describe a repair technique beginning with extension of the tear into the surrounding tissue [13, 17], two of which created a fourth-degree tear; the other extended the episiotomy to include the rectal tear leaving the anal sphincter intact to improve visibility [13]. In all included cases the rectal mucosa and vaginal skin were repaired separately with dissolvable sutures. Five cases described a three-layer closure, with the ‘perirectal tissue’ [13], ‘perirectal fascia’ [17] ‘muscularis’ [10], ‘musculofibrous perineal body’ [6] or ‘rectovaginal septum’ [3] also repaired. In six cases [3, 12, 13, 17] the rectal mucosa was repaired with interrupted sutures, two using 4–0 Monocryl (poliglecaprone 25) and one using 3-0 Vicryl. Two [10, 13] repaired the rectal mucosa continuously; one reported using 1 Vicryl (polyglactin 910). Another case [6] used 0-Vicryl, but did not state if this was continuous or interrupted. In three cases [3, 12, 17] the vaginal skin was repaired with continuous Vicryl, and in six reports [6, 10, 13] the type of repair was not stated. Two cases reported giving intra-operative intravenous antibiotics and eight cases reported giving antibiotics and laxatives post-operatively. All cases had follow-up, four at 6 weeks or 3 months, with no complications and all patients remained asymptomatic.

### Croydon University hospital case series

Between April 2012 to March 2020 three patients who sustained isolated rectal buttonhole tears were identified (Table 2). During that time there were 21,929 vaginal deliveries giving a rate of 1 per 7310 vaginal deliveries (incidence of 0.014%). Of the three cases one was a ventouse delivery (case 1) and two were forceps (cases 2 and 3). All cases had an associated episiotomy; case 2 had a concurrent 3a tear. Cases 1 and 3 described the defect as 4–5 cm and 3 cm, respectively, and in case 2 the length of the tear was not described. Case 1 was repaired jointly by an obstetrician and colorectal surgeon; the two other cases were repaired by a senior obstetric trainee supervised by a consultant. In all three cases, interrupted 2–0 Vicryl was used to suture the rectal mucosa. One surgeon (case 3) used three layers to close the buttonhole tear, one surgeon (case 1) used two layers, and the other did not specify.

**Table 2:** Case series from Croydon University Hospital

Case number	Mode of delivery	Type of injury	Type of repair	Repair conducted by	Post-operative management	Follow-up and patient symptoms
1	Ventouse	Episiotomy and 4–5 cm of rectovaginal septum, proximal to sphincters	2-layer inverting 2–0 Vicryl. Episiotomy repaired in layers	Colorectal surgeon jointly with obstetrician	5 days antibiotics and Lactulose	Follow-up at 3 months, asymptomatic, endoanal ultrasound normal
2	Forceps, right occiput- posterior	Episiotomy, 3a tear and isolated rectal buttonhole tear	Interrupted 2–0 Vicryl rapide, knots in rectal lumen. 3a tear and episiotomy repaired	Obstetrician	7 days antibiotics, 10 days Lactulose	Follow-up 6 weeks, asymptomatic, endoanal ultrasound normal
3	Forceps, direct occiput-posterior	Episiotomy and 3-cm isolated rectal buttonhole tear	3-layer Interrupted 2–0 Vicryl to mucosa, continuous to muscle (2–0 Vicryl) and vaginal (2–0 Vicryl rapide) mucosa. Re-sutured by consultant	Obstetric trainee (supervised by obstetric consultant)	Vaginal pack, 14 days Lactulose, 3 days antibiotics	Wound breakdown, secondary repair attempted and persistent fistula. Defunctioning ileostomy with further repair

In case 3 forceps were used to deliver the baby in the direct occipito-posterior position. There was evidence of tearing of the posterior vaginal wall after the first pull on the forceps and according to the notes, a buttonhole defect was palpated between contractions. An episiotomy was performed with the second pull and the baby was delivered. After repair of the rectal mucosa a rectal examination was performed to check its integrity. It is then documented that the ‘muscle’ above the mucosa was repaired and the vaginal mucosa was closed. On removal of the vaginal tampon the ‘friable vaginal tissue then tore’ and a repeat repair was performed by the consultant.

In all cases, post-operative antibiotics were prescribed for a minimum of 3 days, as well as laxatives. Cases 1 and 2 were asymptomatic at follow-up with a normal endoanal ultrasound scan. Case 3 suffered a breakdown of the wound at 27 days post-delivery and had a secondary repair by a colorectal surgeon at 62 days after the initial repair. The persisting recto-vaginal fistula was described as a 4 mm opening. During the repair the caudal mucosa was raised with saline; the circular muscle was advanced and opposed with 2–0 Vicryl. The surgeon then performed a mucosal advancement flap, closed with continuous 2–0 Vicryl. On day 20 post-operatively, the patient had a contrast enema which revealed a persistent recto-vaginal fistula. A de-functioning ileostomy with a second repair (transvaginal mucosal advancement flap closed with 2–0 Vicryl) of the rectovaginal fistula was performed at 167 days after the primary obstetric repair at delivery. At the time of writing this article, she still had the ileostomy and was awaiting a further contrast enema.

## Discussion

Our review of literature and the case series highlighted that primary repair, despite some variation in technique, resulted in satisfactory closure of the tear and uneventful recovery in the majority (92%) of cases. However, these data of good outcome must be interpreted with caution because we do not have a denominator and clinicians are unlikely to report cases with adverse outcomes.

In the original description of the classification of perineal trauma by Sultan [2], isolated rectal tears were not considered to be fourth-degree tears unless associated with an OASI. However, as described in this paper, two cases with an isolated buttonhole tear also had an associated OASI. We therefore propose that in keeping with the Sultan Classification [2, 16] when an OASI is in continuity with a rectal tear it should be called a fourth-degree tear. However, when a third- or fourth-degree tear occurs concomitantly with an isolated rectal buttonhole tear (an island of intact mucosa between the third/fourth-degree tear and the isolated rectal buttonhole tear) it is classified and described as such.

**Fig. 2:**
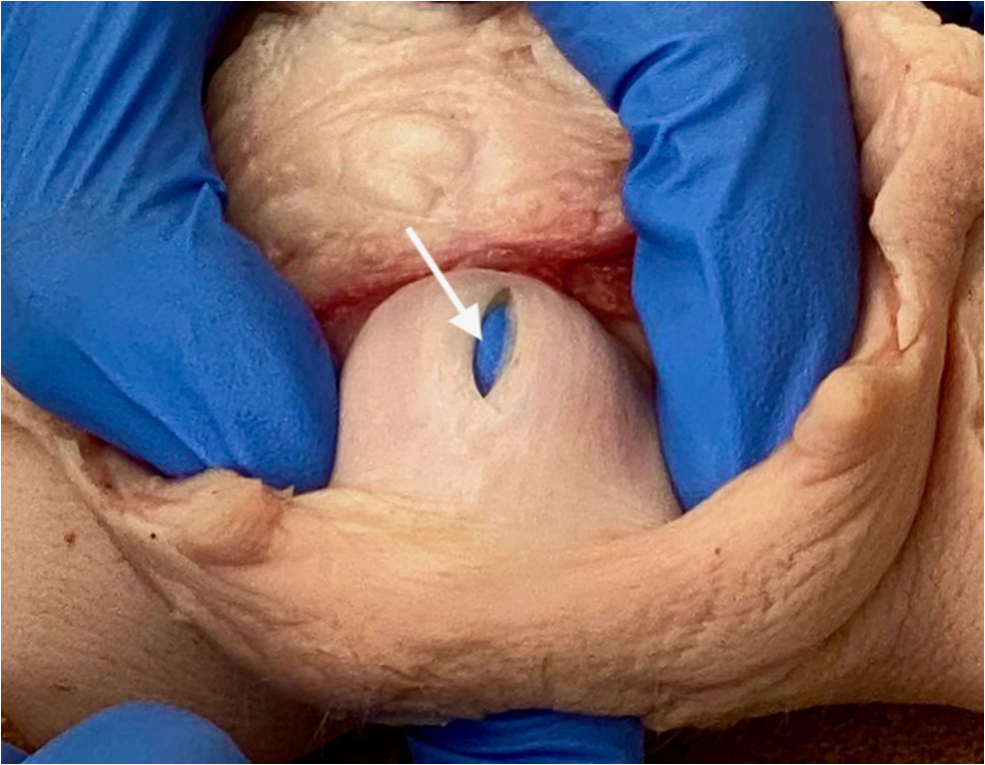
Isolated rectal buttonhole tear in pig specimen (arrow)

If a buttonhole tear is missed or if the repair breaks down, a rectovaginal fistula can persist. Therefore, it is imperative that an accurate diagnosis is made at the time of delivery as the best chance of success is with the first attempt. To avoid missing such injuries a digital rectal examination should be performed as part of the perineal and vaginal assessment following every vaginal delivery [1, 16, 18]. This should be conducted by a trained healthcare professional to exclude OASI and isolated rectal buttonhole tears after all vaginal deliveries irrespective of whether the perineum appears to be intact or not [1] (Figs. 1 and 2). This is also highlighted in the case reports by Diepenhorst, where three cases are described with an apparently intact perineum [17]. A rectal examination is repeated after any suturing to ensure that no suture has been inserted inadvertently into the rectum [4]. As there would not be a palpable knot in the anal canal, any puckering of the rectal mucosa would suggest that there could be a loop of suture material. A combined vaginal and rectal examination with the index finger of each hand performing a pill rolling action would help to identify any suture between the vagina and rectum. In this situation the repair should be un-done and the stitch removed. If the stitch is not removed, tissue necrosis can occur with a resultant rectovaginal fistula typically presenting 7 to 10 days later.

**Fig. 3:**
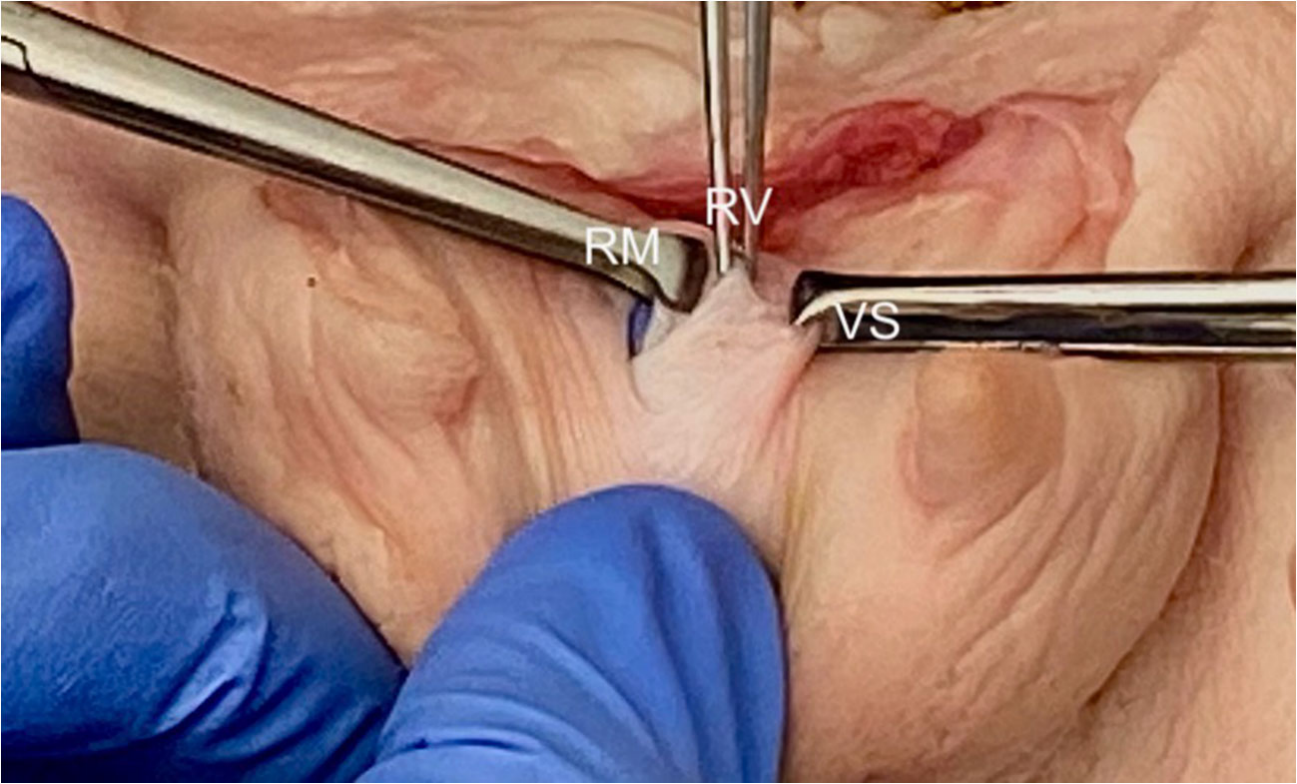
Layers for repair in pig specimen (RM = rectal mucosa, RV = rectovaginal fascia, VS = vaginal skin)

In our case series there was a lack of consistency in the description of the repair techniques with some using a two-layer closure and others using a three-layer closure. The rectovaginal fascia, or rectovaginal septum, is a layer of tissue between the rectum and vagina (Fig. 3) and constitutes the third layer. The rectovaginal fascia should always be repaired to separate the repaired rectal mucosa from the vaginal skin [4]. A three-layer approach is recommended (Table 3, Video 1), when possible, in fistula closure surgery to minimise the risk of recurrence [19]. In some situations where this is not possible, surgeons use other intervening tissue such as omentum or labial fat (Martius graft) [19, 20]. The rectovaginal fascia is also known as the rectovaginal septum. Its presence in females has previously been debated [21]. In a literature review, Dariane et al. discuss papers describing the embryological origin, microscopic description, anatomical location, relations and function of the rectovaginal septum. They concluded that, present from an embryological stage, it is a distinct connective tissue layer between the vagina and rectum. Its function is likely to be trophic support and compartmentalisation of subperitoneal spaces, limiting expansion of infection and tumours [21]. Given these important functions, and the need for an intermediate layer of tissue to minimise the risk of recurrence, care should be taken to identify and repair the rectovaginal fascia separately.

**Table 3:** General principles for repair of obstetric isolated rectal buttonhole injuries

Step	Principle
Surgeon	Repair by an appropriately trained clinician or by a trainee under supervision. As these injuries are so rare, a consultant should always be present. If the obstetric consultant is not confident it should be performed jointly with a colorectal surgeon.
Colorectal opinion	Should always be sought for high rectal buttonhole tears (> 7 cm from the anal verge or if there is faecal soiling). A covering colostomy is rarely performed but may be considered in these scenarios. The risks and benefits of colostomy should be discussed with the patient.
Setting	Repair should take place in an operating theatre, under regional or general anaesthesia, with good lighting and appropriate instruments.
Examination	A systematic digital vaginal and rectal examination must be performed to exclude any additional injuries and in particular an OASI should be excluded (Fig. 2).
Prior to repair	The proximal and distal end of the rectal laceration must be clearly identifiable before suturing. The three layers for repair (rectal mucosa, rectovaginal fascia and vaginal skin) must be identified (Fig. 3).
General principle	Figure-of-eight sutures should be avoided because they are haemostatic in nature and may cause tissue ischaemia.
Repair of rectal mucosa	Using a transvaginal approach, a non-locking continuous 3–0 polyglactin suture, with knots on the vaginal side of the rectal tear. Perform a rectal examination to confirm that good apposition of the mucosa is obtained.
Repair of rectovaginal fascia	Using an interrupted mattress technique using a 2–0 or 3–0 PDS sutures.
Repair of vaginal skin	Continuous non-locking 2–0 Vicryl sutures.
Following repair	A digital vaginal and rectal examinations should be performed to ensure complete closure of the tear.
Complex tears	If the distal end of the anorectal mucosal tear is not clearly identifiable, it will be the only indication to create a 4th-degree tear by cutting through the intact anal sphincters and anorectal mucosa to meet up with the distal end of the rectal buttonhole tear. Repair is then performed as described for a 4th-degree tear [4].
Antibiotics	Intra-operative broad-spectrum intravenous antibiotics should be given and continued as per local protocol. Our practice is to continue oral antibiotics for at least 3 days.
Laxatives	A stool softener, such as Lactulose, should be prescribed for at least 10 days.
Follow-up	Arranged in 6 weeks or earlier if indicated.

In terms of suturing methods and types of sutures described there are some discrepancies. Two cases describe laying the knots of the sutures in the rectal lumen. When repairing a fourth-degree tear, knots can be either in the rectal lumen or a subcuticular repair of the anal epithelium via the transvaginal approach [4]. The practice of tying the knots in the rectal lumen dates to when only chromic catgut was available. It is known that this suture causes tissue reactions because it dissolves by phagocytosis and hence predisposes to infection when the suture material is present within tissues [4]. Unlike with a fourth-degree tear, access to the rectal lumen in a buttonhole tear is difficult and therefore it will not be possible to suture the full length of the tear from the proximal to the distal end with sutures in the rectal lumen. Therefore, it is best to repair the rectal mucosa with a continuous suture with the knots tied on the vaginal aspect (Table 3, Video 1).

Suture material used for repair is similar in all cases. For the rectal mucosa, Vicryl (polyglactin 910), an absorbable, synthetic, braided suture, was most commonly used. We recommend a continuous Vicryl 3–0 suture to repair the rectal mucosa, similar to that recommended for a fourth-degree tear, as it causes less rectal irritation than a Polydiaoxanone (PDS) suture [14] (Table 3). The repair of the rectovaginal fascia was mentioned in six cases, three of which used Vicryl, one used Monocryl and two did not name the suture. The vaginal skin was sutured with Vicryl in all cases where sutures were named. A 2–0 or 3–0 PDS suture is recommended to repair the rectovaginal fascia to give support to this structure and continuous 2–0 Vicryl for the vaginal skin (Table 3, Video 1).

Intra-operative broad-spectrum antibiotics were given in eight cases and this is in keeping with the practice following OASI [4, 22]. There is little evidence to support the use of post-operative antibiotics in OASI and therefore this should be done in accordance with local advice from microbiologists [16]. Ten (83%) of the cases describe giving post-operative antibiotics. Two of the cases do not describe the antibiotic course duration. In those cases that mentioned a duration, it ranged from 3 to 7 days. Given the location of the tear into the rectal mucosa, it is an area with a high risk of infection and therefore prophylactic antibiotics during healing could potentially reduce the risk of infection.

It is important to avoid disruption of the sutured mucosa due to faecal impaction caused by constipation. Accordingly, laxatives were given in most cases. After an OASI, laxatives are given post-operatively to reduce straining and faecal impaction and the same should be done for rectal buttonhole tears (Table 3).

Morrell et al. and Diepenhorst et al. describe three similar cases in which the buttonhole tear was extended to improve the visibility of the operating field [13, 17]. In two cases, it was extended to cut through the sphincter to create what appeared to be a fourth-degree tear. This method is sometimes used to repair rectovaginal fistulas [23]; however, it is not recommended for acute obstetric tears because of the risk of anal dysfunction, even with an adequate repair [19]. The only reason for extending a buttonhole tear to a fourth-degree tear is if the distal end of the tear cannot be visualised and therefore it will not be possible to perform an adequate repair of the anorectal mucosa. This, in turn, may predispose to the development of a persistent fistula.

Only one of the reported cases did not result in closure after the primary repair. The reason for the breakdown of the primary repair is unclear. This case was complicated by additional vaginal tears, and part of the initial repair by the trainee had to be re-sutured by the consultant. In comparison to the other included cases, this was one of only five cases to mention a three-layer repair and also where the fewest days of post-operative antibiotics were given (3 days compared to 5 and 7 days, in the other two Croydon cases). However, other factors to consider include perineal hygiene and constipation. It is interesting to note that, in this case, during the first 14 days after the initial repair the dose of laxatives was increased. It is possible that she could have been very constipated, and straining could have contributed to disruption of the repair.

All cases were followed up clinically, with four cases having an endoanal ultrasound scan. A post-operative review is essential to exclude symptoms and signs of a rectovaginal fistula. Similar to an OASI, follow-up should be at a minimum of 6 weeks. No subsequent pregnancy was recorded in any of our cases. We recommend an antenatal review, during a subsequent pregnancy, to enquire about any symptoms of anal incontinence and perform endoanal ultrasound to exclude any unidentified sphincter damage. If anal sphincter defects are identified, we follow the protocol described by Jordan et al. [24]. If the sphincter is intact, evidence is lacking to suggest the appropriate mode of delivery. Given that the cause of the rectal buttonhole tear in most cases remains unknown, and the risk of recurrence has not been established, the choice of a caesarean section should rest with the woman after an informed discussion of the pros and cons of a vaginal delivery.

If a rectovaginal fistula is detected at follow-up it is known that approximately 50% of small obstetric recto- and anovaginal fistulae can heal spontaneously [25]. Rectovaginal fistulae are difficult to repair, have a high recurrence rate [26] and can lead to the need for a stoma to defunction the bowel during healing [8]. A diverting stoma is rarely indicated in acute OASI unless the tear is large and extends above the pelvic floor, or there is faecal contamination [4, 19].

Although there is a paucity of evidence in the literature about primary repair of obstetric isolated rectal buttonhole tears, there are many publications on repair of rectovaginal fistulae [5, 8, 20, 26]. There are several alternative techniques describing repair of rectovaginal fistulae although there is no consensus on the ideal repair [8]. Rectovaginal fistula can be caused by obstetric injury, congenital malformation, trauma (some from sexual violence), perianal sepsis, Crohn’s disease, during another surgical procedure or treatment with radiation for malignancy [5]. Surgical options for repair include muscle transposition, plugs, fistula excision or even laparotomy [5, 8, 27]. Reisenauer describes closure of obstetric rectovaginal fistulae using the transvaginal route with excision of the epithelialised fistula, haemostasis and tension-free closure of the fistula in several layers [28]. Forty-two percent of the patients in this study also had a de-functioning stoma at the time of fistula repair [28]. By contrast, a de-functioning stoma is very rarely indicated during repair of acute OASIs [4].

Hauch et al. describe many cases of secondary rectovaginal fistula repair and propose that supra-sphincteric fistulae are repaired by laparotomy with muscle transposition (rectus abdominus) or omental interposition [8]. For inter-sphincteric or low fistulae a perineal approach is preferred. Rectovaginal fistulae that have a healed, epithelialised edge are different from a fresh injury; therefore, although there are similarities, it should not be directly compared in terms of repair techniques. Our case series had complete recovery following primary repair, without faecal diversion in 91% of cases indicating that a stoma is rarely necessary. Based on this, women should be counselled that there is a 5 to 10% chance that they may require a stoma if a complication of a rectovaginal fistula occurs.

The strengths of this review are that we have included all available published cases describing primary obstetric repair of isolated rectal buttonhole injuries and also reviewed cases from our own institution, making it the largest series available.

The limitation to this case series is the relatively small sample size. Given the rarity of an obstetric isolated rectal buttonhole tear and hence fewer publications, it is difficult to obtain a large enough number of cases to analyse outcomes. There is possible reporter bias in the published cases because a case report is less likely to be written for a patient who had a complicated recovery. However, it is known from medicolegal practice that these injuries, that are usually missed, do occur more frequently than reported, highlighting the importance of a structured genital and rectal examination after every vaginal delivery.

## Conclusions

Given the variety of repair techniques used in these case reports, we have provided recommendations of best practice based on our experience of acute perineal and anal sphincter trauma in both developed and low-resource countries. We adhere to surgical principles of fistula surgery, namely identification of the full extent of injury, repair of the tissues without tension, approximation of the laceration with a minimum of three layers and the use of intra- and post-operative antibiotics. Perineal hygiene and avoidance of constipation by taking stool softeners are also important to minimise the risk of wound breakdown. Overall, the success rate of primary repair of rectal buttonhole tears appears to be good and a diverting stoma is rarely indicated. There is insufficient evidence to suggest the appropriate mode of delivery in a subsequent pregnancy. However, as the cause of the rectal buttonhole tear in most cases remains unknown, the choice of a caesarean section should rest with the woman after an informed discussion of the pros and cons of a vaginal delivery.

## Electronic supplementary material

Video 1 Repair of isolated rectal buttonhole tear in pig specimen. (MP4 481644 kb)
